# Real-time auto-adaptive margin generation for MLC-tracked radiotherapy

**DOI:** 10.1088/1361-6560/62/1/186

**Published:** 2016-12-16

**Authors:** M Glitzner, M F Fast, B Denis de Senneville, S Nill, U Oelfke, J J W Lagendijk, B W Raaymakers, S P M Crijns

**Affiliations:** 1 Department of Radiotherapy, University Medical Center Utrecht, Heidelberglaan 100, 3584 CX Utrecht, The Netherlands; 2 Joint Department of Physics at The Institute of Cancer Research and The Royal Marsden NHS Foundation Trust, London, UK; 3 Mathematical Institute of Bordeaux, UMR 5251 CNRS/University of Bordeaux, 33405 Talence Cedex, France; m.glitzner@umcutrecht.nl

**Keywords:** MLC, tracking, margins, error, statistics, real-time

## Abstract

In radiotherapy, abdominal and thoracic sites are candidates for performing motion tracking. With real-time control it is possible to adjust the multileaf collimator (MLC) position to the target position. However, positions are not perfectly matched and position errors arise from system delays and complicated response of the electromechanic MLC system. Although, it is possible to compensate parts of these errors by using predictors, residual errors remain and need to be compensated to retain target coverage. This work presents a method to statistically describe tracking errors and to automatically derive a patient-specific, per-segment margin to compensate the arising underdosage on-line, i.e. during plan delivery.

The statistics of the geometric error between intended and actual machine position are derived using kernel density estimators. Subsequently a margin is calculated on-line according to a selected coverage parameter, which determines the amount of accepted underdosage. The margin is then applied onto the actual segment to accommodate the positioning errors in the enlarged segment.

The proof-of-concept was tested in an on-line tracking experiment and showed the ability to recover underdosages for two test cases, increasing ${{V}_{90 \%}}$ in the underdosed area about $47 \% $ and $41 \% $, respectively. The used dose model was able to predict the loss of dose due to tracking errors and could be used to infer the necessary margins.

The implementation had a running time of 23 ms which is compatible with real-time requirements of MLC tracking systems. The auto-adaptivity to machine and patient characteristics makes the technique a generic yet intuitive candidate to avoid underdosages due to MLC tracking errors.

## 1. Introduction

In radiotherapy, dose conformity, the ratio of actual to intended dose deposition, is impaired by the change of patient anatomy during (*intra*) treatment and between (*inter*) treatment fractions. *Intra-fraction* changes occur predominantly in thoracic and abdominal sites which are directly modulated by breathing excursions (e.g. Moerland *et al* (1994), Plathow *et al* (2004)). Tracking can be used to adapt the treatment beam to a variable tumor position (Ruan *et al* 2011). Recently, on-line multileaf collimator (MLC) control has become available on the treatment machines of major radiotherapy vendors and first tracked deliveries were performed *in vivo* (Colvill *et al* 2015).

**Figure 1.**
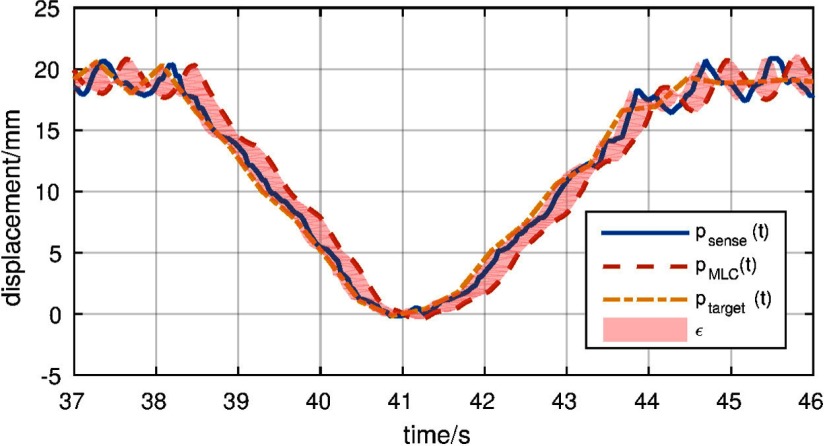
Recorded target (*p*_target_) and MLC (*p*_MLC_) evolution during a tracking experiment. Delayed target positions (*p*_sense_) were used as a feedback variable. The integral tracking errors of the various tracking system components are highlighted in red.

However, the quality of conformity using MLC tracking is to a high extent influenced by the underlying system delay, which can amount up to several hundreds of milliseconds (Hoogeman *et al* 2009, Tacke *et al* 2010, Depuydt *et al* 2011, Fast *et al* 2014, Bedford *et al* 2015, Glitzner *et al* 2015). Typically, the feedback controller in MLC tracking processes the incoming signal of an imaging/positioning modality. Subsequently, the (affine) target displacement is extracted from the signal. A planned reference segment, shifted to the new target position in beam’s eye view (BEV), is then sent to the MLC controller. All of these components exhibit an inherent time delay which cause lag and thus misalignment between the target and the treatment beam. As a simplification, these time delays are usually quantified using sinusoidal motion patterns, assuming a linear phase behavior of the entire MLC system (Glitzner *et al* 2015). In reality, the electromechanic MLC system will not behave according to a single, pre-set lag but will show a response comparable to figure 1. The prescribed position (blue) will not only cause a shifted MLC response (red). Contrary, the machine response will show complex over- and undershoots, which cannot be explained by a constant lag alone.

Look-ahead predictors are designed to compensate the constant lag effects. In general, however, the quality of the predictor strongly depends on the characteristics of the patient motion, such as amplitude, frequency and phase variations, as well as on its parameterization and the machine performance itself (Ruan 2010, Krauss *et al* 2011).

Additionally look-ahead predictors can (by definition) not account for the mentioned non-constant-lag effects. The tracking errors arising due to these imperfections can be regarded as stochastic errors. In order to retain target coverage they have to be compensated e.g. by using tracking margins.

In this study a method to automatically compensate for dosimetric errors arising from machine and physiologic uncertainties using auto-adaptive tracking margins is proposed. In contrast to the margins defined by International Commission on Radiation Units and Measurements (ICRU) (ICRU 2010) which are applied to the clinical target volume (CTV) during the planning process, the proposed method works on a per-segment basis during delivery, i.e. after plan optimization. The real-time process is intended to provide optimal target coverage in the sense of percentual coverage to a selected confidence level. The method uses the capability to read-out the actual MLC positions $\boldsymbol{p}_{{\text{MLC}}}(t)$ in every control system cycle of 40 ms. These are combined with the retrospectively known $\boldsymbol{p}_{{\text{target}}}(t)$ to estimate the positioning error $\boldsymbol{\epsilon}(t)$ and integrate it into a tracking margin.

## 2. Methods

**Figure 2.**
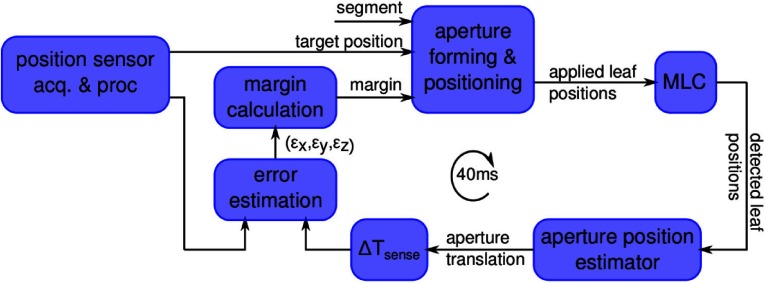
Proposed adaptive tracking margin control system.

The set-up comprises multiple hardware and software components, as depicted in figure 2. Every block in the component diagram is explained in detail subsequently. All software was based on C++ implementations running on a Linux Mint computer (kernel version 3.16) with two Intel Xeon E5-2620 at 2 GHz and 32 GB memory.

### 2.1. Position acquisition & processing

In order to provide the MLC feedback-loop with a reference variable, the target positions are continuously sent by the position sensing module.

This abstracted block can source data of any kind, such as megavoltage imaging (MV), kilovoltage imaging (kV), magnetic resonance imaging (MRI) or marker/transponder information. Eventually, the processing cascade extracts an estimated target position

1 \begin{eqnarray*}\boldsymbol{p}_{{\text{target}}}(t)={{\left(\,{{p}_{x}},{{p}_{y}},{{p}_{z}}\right)}^{T}}(t).\end{eqnarray*}

Position sensing is likely to be the main source of delay in an MLC control system due to the complexity of acquisition and processing of imaging data. In order to keep track of these delays, the pipeline requires thorough timestamping throughout the processing cascade.

The timestamp of $\boldsymbol{p}_{{\text{target}}}(t)$ is assumed to be adjusted by the target position’s acquisition and processing delay $ \Delta {{T}_{\text{sense}}}$. Although some jitter has to be expected, it is assumed to be minimal on real-time implementations; thus $ \Delta {{T}_{\text{sense}}}$ is set to constant values for this proof of concept. Generally, however, the capability of processing non-constant delays can be implemented easily by dynamically adapting the interpolation kernels’ shift (*look-back*).

### 2.2. Aperture forming & positoning

In this block, a valid MLC prescription is generated, which comprises position data for the leaves and jaws of the MLC.

In general, segment shapes from the treatment plan and actual target positions are passed on to this block. The planned aperture is consequently shifted to the new target position, incorporating the imposed discretization by the MLC leaves. In this work, an implementation based on Sawant *et al* (2008) was employed. This algorithm subdivides the coarse leaf-width into subleaves and translates the initial aperture according to the finer discretization. Upon prescription of a polygon, the leaf-positions are determined by averaging over the subleaf-positions. The diaphragms were steered as in Fast *et al* (2014), applying the offset perpendicular to leaf-travel direction directly to the planned jaw-positions.

In this work, the aperture forming and positioning was extended to impose segment margins in real-time. In order to do so, the process receives margin prescriptions in the form

2 \begin{eqnarray*}\boldsymbol{m}(t)={{(({{m}_{\hat{x}+}},{{m}_{\hat{x}-}}),({{m}_{\hat{y}+}},{{m}_{\hat{y}-}}))}^{T}},\end{eqnarray*}

$\hat{x}$ and $\hat{y}$ denoting the axis parallel and perpendicular to the leaf-travel direction, respectively. The side, on which the margin is added on the respective axis relative to the MLC’s isocenter is indicated with  +  and  −. This enables prescriptions of individual margins for both sides of both the principal axes of motion.

**Figure 3.**
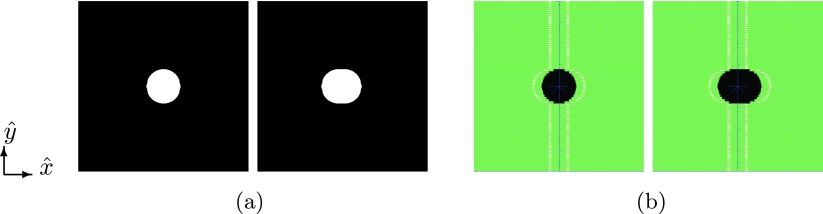
(a) shows the effect of margin-controlled dilation on rasterized polygons; the original aperture (left) is dilated in $\hat{x}$-direction. From the contours of the dilated shape, a new valid MLC-segment is calculated by the leaf-shaping algorithm (b). (a) Rasterized polygon segments. (b) Prescribed segments.

The tracking margins were calculated and imposed on the planned segment using the following sequence: first, the planned segment is transformed into a polygon which in turn is rasterized using the OpenCV library (Bradski 2000). Rasterization yields a grid with an isotropic resolution of 0.25 mm in both leaf travel and leaf count direction. Secondly, using morphologic dilation in the two principal directions ($\hat{x}$ and $\hat{y}$), an expanded raster is created. Using OpenCV, the vertices of the raster’s outline are determined and translated into a polygon (see figure 3). The resulting polygon is then applied to the MLC using the leaf-shaping algorithm. The spacing of the MLC’s diaphragms is modified similarly, using the margins perpendicular to the leaf travel direction.

The dilation kernel is calculated using the grid resolution of the margin generator and the margin size in the individual directions. At a grid resolution of 0.25 mm, a typical kernel element would, assuming a margin of (−4 mm, 2 mm), contain 25 elements: 16 in negative direction, one center element and 8 in positive direction.

### 2.3. Error quantification

Once an aperture is applied to the MLC, the on-board control system drives the leaves and jaws in order to reach the new position in a time frame of 40 ms.

Due to mechanical inertia, the MLC-system will answer to a prescribed position change with delay. This change can be described with an MLC-latency $ \Delta {{T}_{\text{MLC}}}$ for sinusoidal reference signals (Glitzner *et al* 2015)

3 \begin{eqnarray*}\boldsymbol{p}_{{\text{MLC}}}(t)=\boldsymbol{p}_{{\text{sense}}}\left(t- \Delta {{T}_{\text{MLC}}}\right),\end{eqnarray*}

with $\boldsymbol{p}_{{\text{MLC}}}(t)$ and $\boldsymbol{p}_{{\text{sense}}}(t)$ being the center of gravity (COG) positions of the actual and the ideal aperture, respectively.

However, tracking physiologic motion is more complex. Thus a $ \Delta {{T}_{\text{MLC}}}$-parameterization obtained by the phase difference between two sine curves is insufficient and impossible to extract for a general case. In order to determine the error due to MLC-latency, the actual MLC-position is read out every control system cycle (CSC) (40 ms) using the MLC control system. Neglecting the latency of this readout, the difference to the prescribed position $\boldsymbol{p}_{{\text{sense}}}$ determines the actual tracking error

4 \begin{eqnarray*}\boldsymbol{\epsilon}^{{\prime}}(t)=\boldsymbol{p}_{{\text{MLC}}}(t)-\boldsymbol{p}_{{\text{sense}}}(t).\end{eqnarray*}

In addition, the discussed signal acquisition and processing latency $ \Delta {{T}_{\text{sense}}}$ has to be taken into account into account. The sensed position is considered as a shifted version of the actual target position in the BEV, which reads

5 \begin{eqnarray*}\boldsymbol{p}_{{\text{target}}}(t)=\boldsymbol{p}_{{\text{sense}}}(t+ \Delta T),\end{eqnarray*}

with $\boldsymbol{p}_{{\text{target}}}$ being the real target position at time *t*. The tracking error including $ \Delta {{T}_{\text{sense}}}$ thus reads

6 \begin{eqnarray*}\boldsymbol{\epsilon}(t)=\boldsymbol{p}_{{\text{MLC}}}(t)-\boldsymbol{p}_{{\text{target}}}(t)=\boldsymbol{p}_{{\text{MLC}}}(t)-\boldsymbol{p}_{{\text{sense}}}\left(t+ \Delta {{T}_{\text{sense}}}\right)\end{eqnarray*}

Considering causality, $\epsilon (t)$ is only known $ \Delta {{T}_{\text{sense}}}$ after its occurrence

7 \begin{eqnarray*}\boldsymbol{\epsilon}\left({{t}^{\prime}}- \Delta {{T}_{\text{sense}}}\right)=\boldsymbol{p}_{{\text{MLC}}}\left({{t}^{\prime}}- \Delta {{T}_{\text{sense}}}\right)-\boldsymbol{p}_{{\text{sense}}}\left({{t}^{\prime}}\right).\end{eqnarray*}

Thus, for a given time ${{t}^{\prime}}$, the sensed object position is compared to a shifted MLC-position, yielding an estimated error for that moment.

The errors were measured and extracted independently for each principal direction $\hat{x}$ and $\hat{y}$.

### 2.4. Error statistics

**Figure 4.**
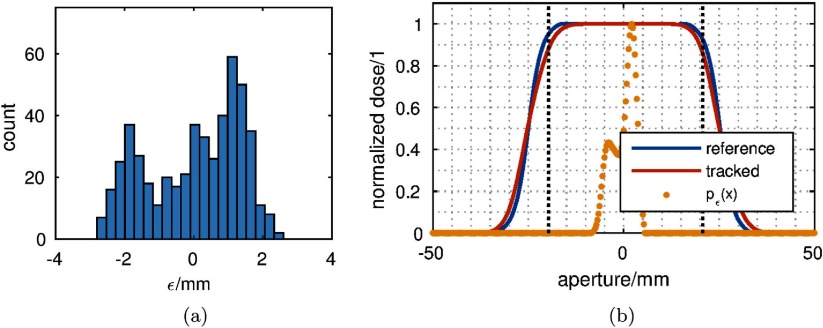
(a) Exemplary aperture positioning error (*ϵ*) histogram for physiological motion. (b) Shows the degraded profile (red) due to the error distribution (yellow). The dashed lines indicate $\hat{x}$, where $D\left(\hat{x}\right)=0.9$. (a) Discrete histogram. (b) Smearing.

The estimated error $\boldsymbol{\epsilon}(t)$ is recorded over an adjustable time period to build up the statistics, necessary to extract statistical features for quantifying an error margin. Figure 4(a) displays a representative *ϵ*-distribution of a tracking experiment using a physiologic tracking variable. The histogram shows the apparent skewness of the multi-modal distribution.

Since histograms inherently suffer from binning uncertainties, kernel density estimators (KDEs) were used to construct continuous densities ${{p}_{\epsilon}}(x)$ from the sample population. KDEs work as a sum of primitive kernels ${{K}_{\sigma}}$ (Elgammal *et al* 2001) centered about each measured sample ${{\epsilon}_{n}}$ as

8 \begin{eqnarray*}{{p}_{\epsilon}}(x)=\underset{n=0}{\overset{N}{\sum}}{{K}_{\sigma}}\left(x-{{\epsilon}_{n}}\right).\end{eqnarray*}

The technique has been already employed in MLC tracking target prediction by Ruan (2010). As a primitive, a zero-mean Gaussian kernel was chosen, which needed to be parameterized by its *σ*, i.e. its bandwidth, which was calculated using a rule-of-thumb (Bowman and Azzalini 1997). As the control system receives updates of the actual target and aperture position every 40 ms, a (cyclic) first in first out (FIFO) buffer of typically *N*  =  500 is updated concurrently and used to populate equation (8). Accordingly, an error statistics of the past 20 s is established, which is then used as an estimate of the current tracking error.

### 2.5. Dose model

In this work, the geometric error statistics are integrated into a dose model to extract a margin description, which is able to compensate tracking errors to a pre-defined extent and can be used as an input for the aperture adaptation algorithm proposed in section 2.2.

For each axis, tracking error statistics ${{p}_{\epsilon}}$ can be translated into dose errors by convolution with the reference dose *D*_ref_

9 \begin{eqnarray*}{{D}_{\text{dyn}}}(x)=\left({{D}_{\text{ref}}}\ast {{p}_{\epsilon}}\right)(x)={\int}_{-\infty}^{+\infty}{{p}_{\epsilon}}\left(x-{{x}^{\prime}}\right)\cdot {{D}_{\text{ref}}}\left({{x}^{\prime}}\right)\text{d}{{x}^{\prime}}\end{eqnarray*}

in analogy to studies about static treatment beam and moving anatomy (Beckham *et al* 2002, Bortfeld *et al* 2004). While, therein, the MLC-segment remained static, both segment and (tracked) anatomy are under motion in this work.

### 2.6. Reference dose model

Ideally, ${{D}_{\text{ref}}}(x)$ in equation (9) is a static, rectangular dose distribution ${{D}_{\text{static},\text{ideal}}}$. However, beam limiting devices such as MLCs do not have ideal cut-off behaviour at segment limits, but exhibit a continuous roll-off, i.e. the penumbra.

To account for the effect in the dose model, the penumbra has to be described and estimated. Similar to convolution-adapted ratio-tissue-air ratio (CARTAR) of Low *et al* (1995), the penumbra due to scatter of beam limiting devices is estimated as a penumbra-generating kernel (PGK). It is assumed to be invariant to shifts with respect to the isocenter and radially symmetric.

**Figure 5.**
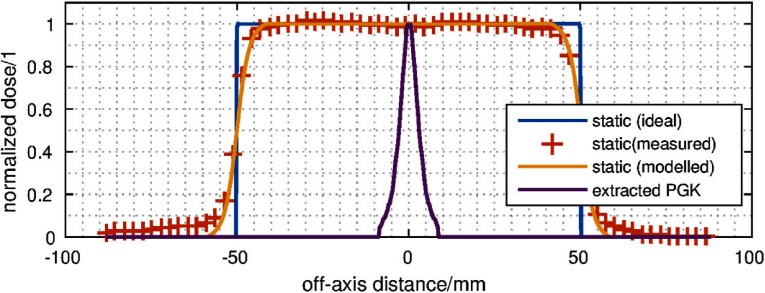
Schematic of penumbra extraction: the PGK (purple) was extracted from the ideal static dose profile (blue) and the measured static dose profile (red, +). This results in a model description of the real static dose profile (yellow).

As depicted in figure 5, the PGK is used to generate ${{D}_{\text{static},\text{real}}}$. It modifies an ideal rectangular (block) dose ${{D}_{\text{static},\text{ideal}}}(x)$, such that

10 \begin{eqnarray*}{{D}_{\text{static},\text{real}}}(x)=\left(\text{PKG}\ast {{D}_{\text{static},\text{ideal}}}\right)(x).\end{eqnarray*}

It is possible to deconvolve a static measurement ${{D}_{\text{static},\text{meas}}}(x)$ with the synthetic ${{D}_{\text{static},\text{ideal}}}(x)$ to obtain $\text{PKG}(x)$. To do so, $\mathcal{F}\left\{{{D}_{\text{ref},\text{ideal}}}(x)\right\}$ needs to be conditioned. Here, we used waterlevels (Richard *et al* 2013) for regularization of $\mathcal{F}\left\{{{D}_{\text{static},\text{ideal}}}(x)\right\}$.

The PGK was estimated accordingly by deconvolution of a calibration film measurement of a $10\times 10$ cm^2^ square field. Subsequently, the extracted PGK was loaded into the tracking software and used for the margin extraction described in the following section.

### 2.7. Margin extraction

Using the dose model of section 2.5, a relation can be established between the reference dose and the dose distribution which is actually being delivered considering the (known) error probability.

By calculating both error-imposed and intended dose distributions, a geometric difference between the two distributions can be estimated. The dose-level at which this difference is estimated is denoted *confidence level* $\hat{D}$ and describes the relative level of reference dose, the control-loop will aim to recover in order to compensate the loss of dose due to tracking errors.

**Figure 6.**
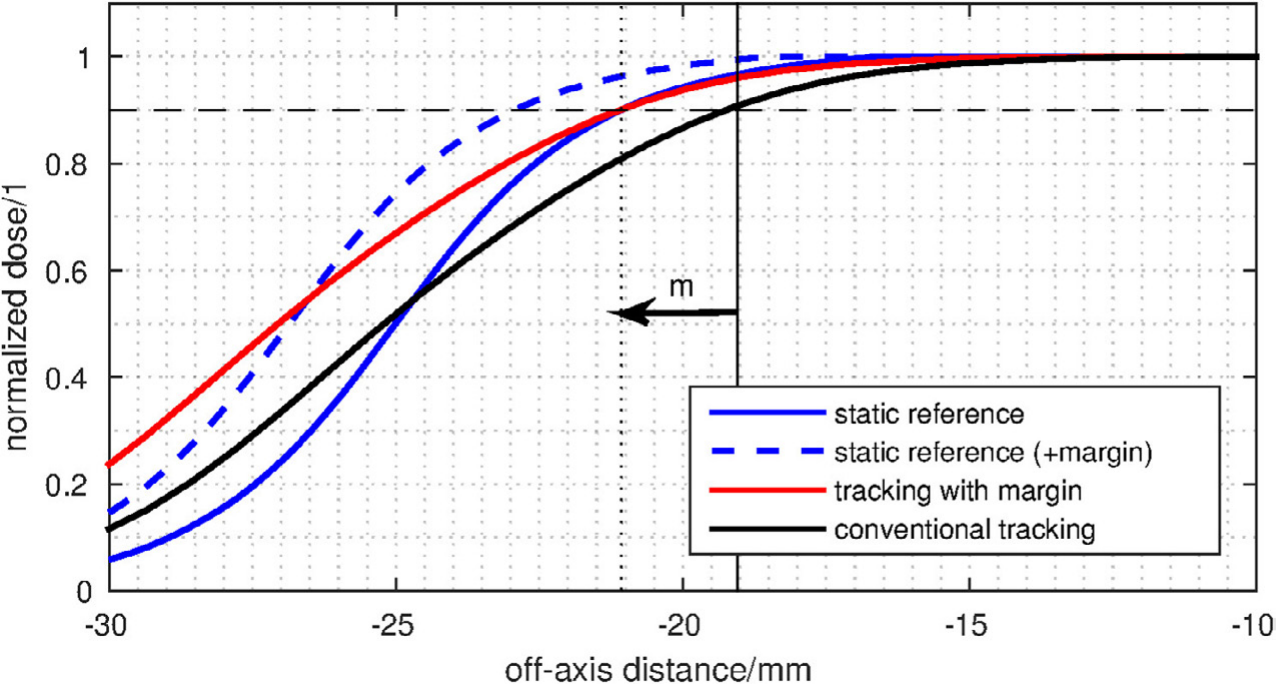
Margin expansion in tracked distribution: tracked delivery causes a loss of dose in the shoulder of the reference distribution. This can be observed in the transition of the static dose line (solid blue) to the tracked dose line (black). In order to compensate this loss to a defined confidence level (90% of intended dose, dashed horizontal line), the initial segment has to be expanded about a margin *m*, indicated by the arrow.

In figure 6, the confidence level is set to $\hat{D}=0.9$. The expansion widths in the same figure is found by solving

11 \begin{eqnarray*}{{D}_{\text{ref}}}(x)={{D}_{\text{dyn}}}(x+m)=\hat{D}\end{eqnarray*}

for *m*. Subsequently, the margin is applied by dilating the static reference aperture about *m*, i.e. the difference between ideal and real dose. In the example of figure 6, *m* equals approximately 2 mm.

### 2.8. Experimental set-up

#### 2.8.1. Film dosimetry.

The performance of the margin compensation was measured using radiosensitive film (Gafchromic EBT3, Ashland, NJ, USA). To calibrate the film, depth-dose curves were acquired. The beam was parameterized with 6 MV, a dose rate of 550 MU s^−1^ and an aperture of $10\times 10$ cm^2^ at isocenter. The gantry was set to 90°, irradiating the horizontally oriented film (aligned with the beam axis) which was sandwiched between two 5 cm square blocks of solid water at 100 cm surface distance from the source. Depth-dose curves of four doses (50, 150, 400 and 700 MU) were exposed to capture a wide dynamic range of the film. After exposure the films were scanned using an Epson Expression 11000XL (Seiko Epson Corp., Nagano, Japan) in transmission mode with 96 dpi spatial resolution and 48 bit color depth (16 bit per channel). Film calibration was done using a fit to a previously measured dose-depth-curve using in-house developed software of The Royal Marsden, London, UK. A one-channel calibration using the green color channel was chosen because it yielded the best fit.

#### 2.8.2. Motion set-up.

For the tracking experiment, the imaging plane was at 100 cm source-to-imager distance (SID). The radiosensitive film was placed under 2 cm of solid water build-up and on 5 cm solid water backscatter material. A 5 cm circular aperture was applied as a reference segment. The gantry was set to 0° and the collimator angle was 90°. The film was irradiated with 550 MU with a 6 MV beam at 550 MU min^−1^.

The physiologic motion data was obtained from the imaging data of two volunteers. The volunteers underwent fast 2D MRI for 5 min, yielding coronal magnetic resonance (MR) images with 10 Hz temporal and $2\times 2$ mm^2^ spatial resolution and 6 mm slice thickness.

**Figure 7.**
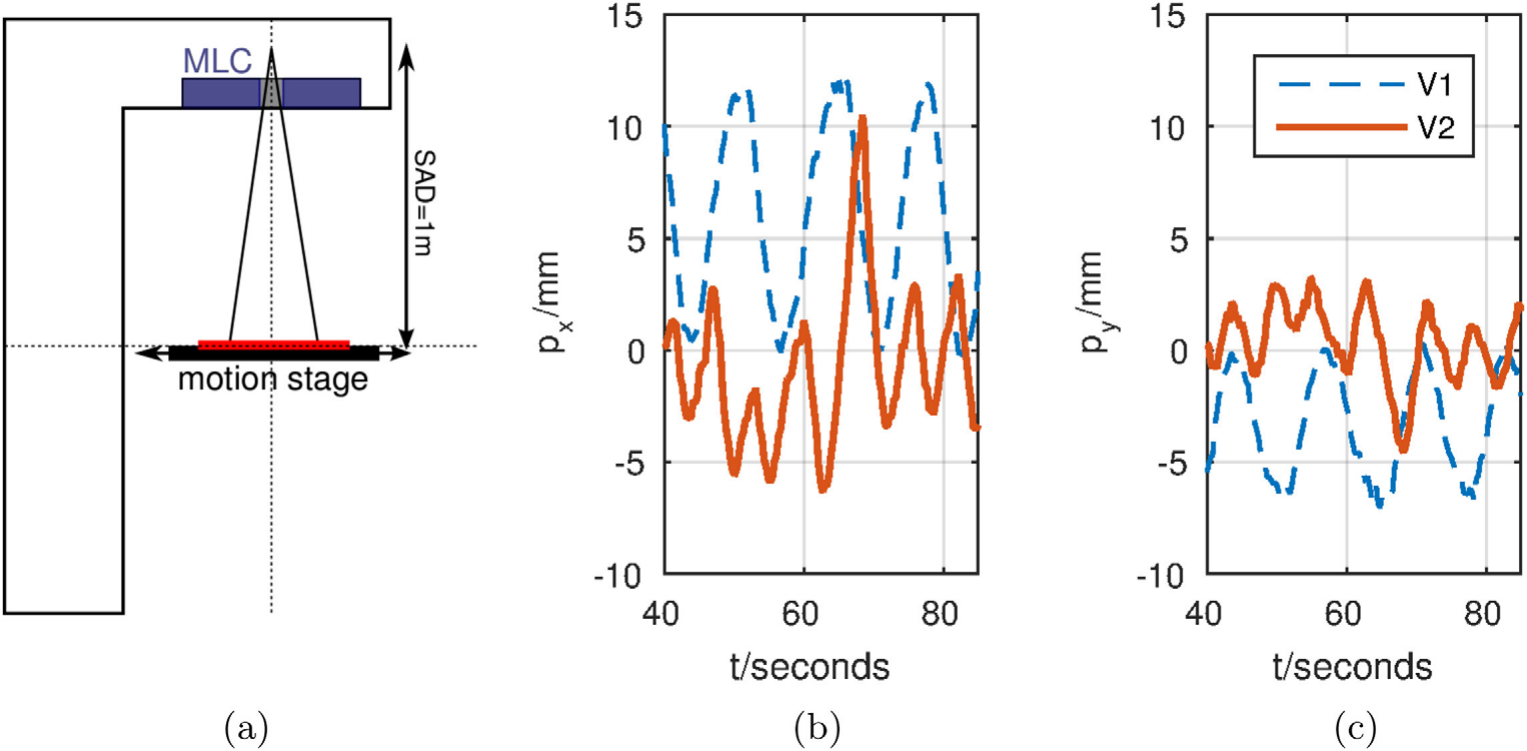
(a) shows the measurement set-up with MLC (purple), motion stage (black) and film set-up (red). (b) and (c) display the *x*- and *y*-components of the motion traces of two volunteers (*V*1 and *V*2) applied to the motion stage. (a) Hardware placement. (b) *x*-displacement. (c) *y*-displacement.

To extract the motion information, the image dynamics were then non-rigidly registered to the first image in the series (reference image) using the method described by Zachiu *et al* (2015). Consequently a point located in the liver dome of each volunteer was selected from the deformation vector field (DVF) to obtain a single motion trace (figures 7(b) and (c)). A point in the liver dome was selected to obtain a challenging target for the MLC tracking system, with displacements stemming from the highly modulating breathing excursions and heart beat. The extracted point served as a motion surrogate for the MLC tracking. This 2D displacement was applied to the MLC using the aperture positioning algorithm of section 2.2.

No breathing instructions were given to the volunteers at any time. The amplitude of motion was comparable to previously reported displacements of the diaphragm (Korin *et al* 1992). However, the volunteers featured different classes of breathing excursion. While *V*1 showed sleep-related regular displacements, *V*2 showed a pattern irregular in frequency and amplitude. The 10 Hz motion traces were upsampled to 64 ms intervals using a linear interpolation kernel in matlab (*The Mathworks*, Natick, MA, USA).

An in-house 4D motion stage (Davies *et al* 2013) was used to move the radiosensitive film, simulating patient motion at 1 m source-to-axis distance (SAD). In addition to executing the motion pattern, the motion stage also provides a position feedback (≈1 ms latency (Fast *et al* 2014)) signal with 30 Hz update rate, which is used as tracking variable.

#### 2.8.3. On-line measurements.

To test the adaptive tracking margin generation and its dosimetric gain, the position feedback was artificially delayed, simulating the latency of a realistic imaging system, comprising acquisition, processing and transmission of imaging data. A latency of 300 ms was thus set for both volunteer trajectories. Accordingly, $ \Delta {{T}_{\text{sense}}}=300$ ms was constantly set for estimating the tracking margin (figure 2). Throughout, coverage values of $\hat{D}=0.9$ were tested and delivered on-line on an Elekta Synergy (Elekta AB, Stockholm, Sweden) research linac. The real-time software controlled the equipped Agility MLC using a research tracking interface provided by Elekta Ltd., UK.

#### 2.8.4. Film analysis.

For each volunteer, four films were irradiated to capture the static, untracked, tracking and margin-compensated tracking case. The doses of all films were referenced to the average dose value in a $15\times 15$ mm^2^ area in the central plateau of the respective static exposure. For qualitative analysis, difference maps between untracked, tracked and (static) reference dose distributions were generated.

To show the compensation performance of the margin generator in 2D, contour lines at $\hat{D}=0.9$ of the static refernce, tracking and margin-compensated tracking exposures were calculated. In 1D, a profile was sampled along the principal axes of motion of the breathing trajectories. The principal axes of motion were extracted from the untracked exposures using Matlab’s (The Mathworks, Natick, MA, USA) principal component analysis (PCA).

Quantitatively, dose-area histograms (DAHs) were generated to measure the dose recovery. To measure the dose recovery performance in the entire circular aperture, an area defined by lower dose threshold of $D&lt;\hat{D}=0.9$ was selected. To specifically select the shoulder area of the 2D dose distributions, thresholds of $\hat{D}&lt;D&lt;0.97$ were chosen. In the selected areas, ${{A}_{90 \%}}$, the relative area with more than $\hat{D}=0.9$, was calculated.

## 3. Results

**Figure 8.**
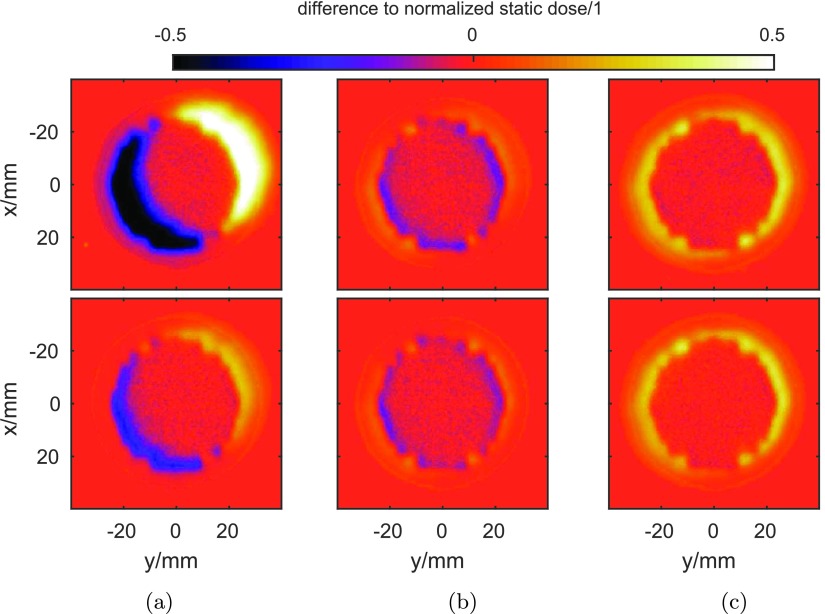
2D dose distributions for two volunteer datasets (upper row and lower row, respectively) relative to the static reference. (a) displays the untracked case. In (b), tracking responses are shown. (c) Shows results with margin expansion.

The dose responses from the tracking experiments with imposed imaging delay of 300 ms are displayed in figure 8. Figure 8(a) illustrates the mean position of the two motion traces, with respect to the reference position. While the first volunteer shows a mean position in the upper right, the mean position of the second motion trace is close to the static reference profile.

The quality of standard tracking is depicted in figure 8(b). It is obviously dependent on the variation of the displacement (see figures 7(b) and (c)). The blue–yellow halo at the outline of the intended (static) dose is the 2D analogy of tracking errors introduced in section 2.4. Yellow zones correspond to an overdosage outside the target, while blue areas mark the critical underdosages compared to the reference profile, which are to be alleviated. With a coverage measure of $\hat{D}=0.9$, these critical underdosages could be covered. This effect is qualitatively shown in figure 8(c). The cost of the increased coverage is an added overdosage outside the target area.

**Figure 9.**
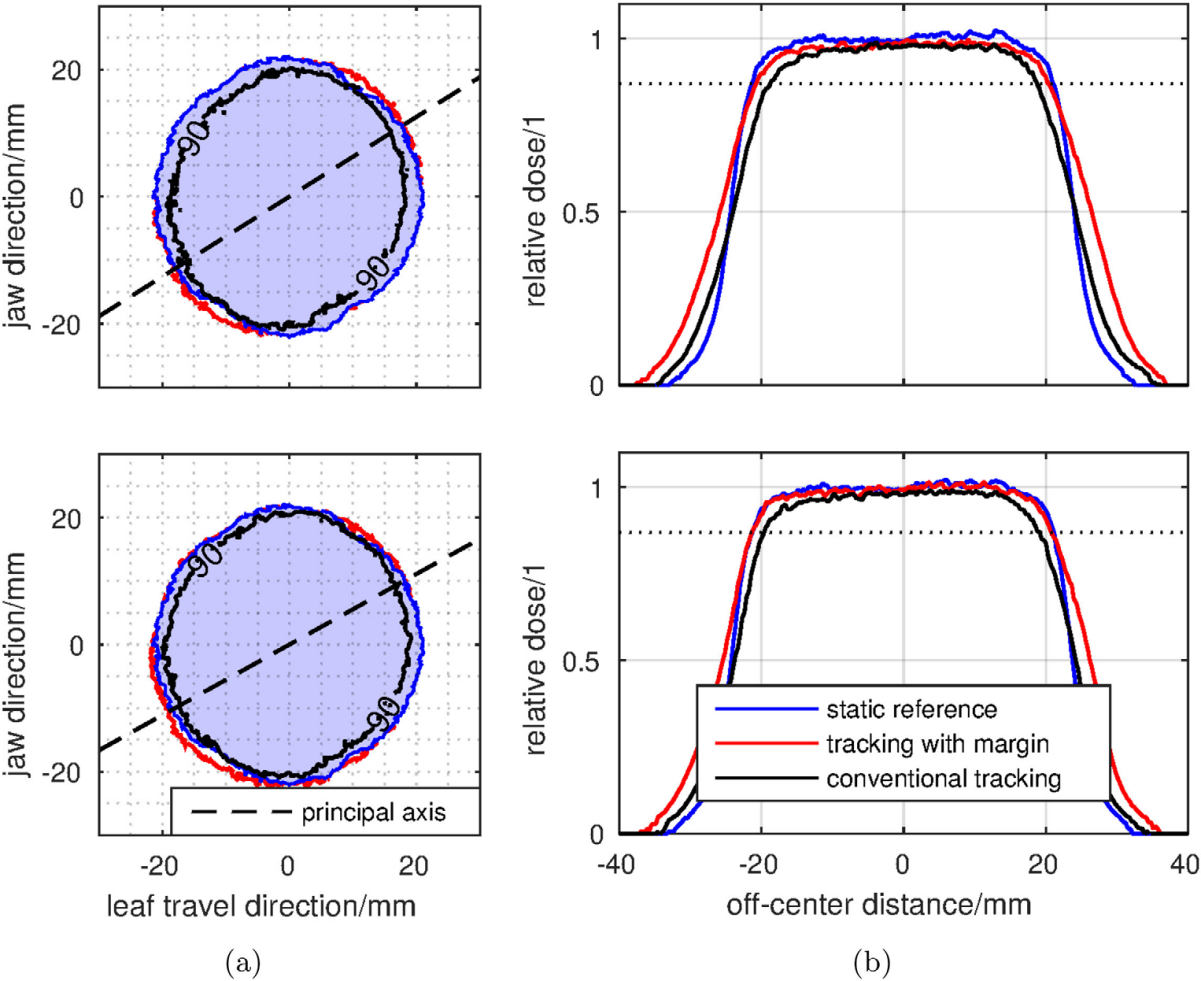
1D/2D-dose profiles: left column (a) shows the 90% contour line of the dose profiles in the case of reference (blue), tracked (black) and tracked with margin (red). The dashed lines show the principal motion axes. (b) Displays the 1D profiles extracted from this. The dotted line indicates the 90% dose level.

In order to test the coverage quality with respect to $\hat{D}=0.9$, 90%-contour lines from the doses of the test subjects are displayed in figure 9(a). The intended overlap between the 90% line of static reference and the margin-expanded tracked case is met in both cases. Deviations from this overlap can be observed at the intersection of an axis at 45°, which are likely caused by imperfect leaf-shaping due to the discrete size of MLC-leaves.

In figure 9(b), dose profiles along the axis of principal motion are displayed. As predicted by the dose model, the dose loss in the shoulders of both distributions could be compensated towards the selected confidence level. Spatial deviations between the 90% lines of static and margin-compensated tracking case are possibly caused by differences in output factor due to the moving aperture. These differences are especially visible in the profile of the first subject (figure 9(b), top).

**Figure 10.**
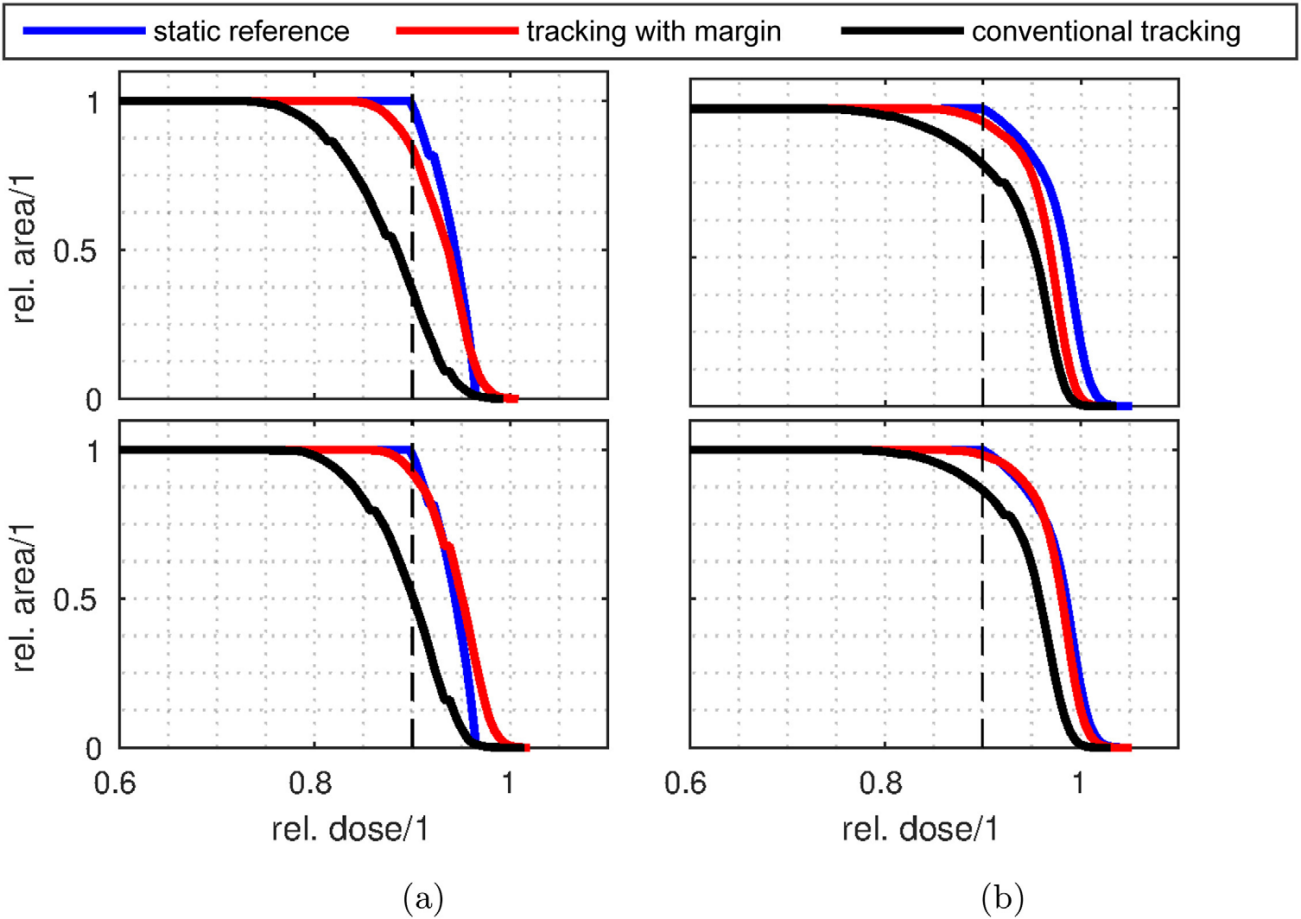
DAH of both test cases (top and bottom row): (a) depicts the DAH-statistics in the shoulder of the dose distribution. (b) displays the DAH calculated over the area with reference dose $D&gt;\hat{D}$. (a) $\hat{D}&lt;D&lt;0.97$. (b) $\hat{D}&lt;D$.

Figure 10(a) shows the DAH in the shoulder of the 2D distribution above $\hat{D}$ of the reference dose distribution. Here, the amount of underdosage due to tracking errors is most crucial. Ideally, the margin-expanded tracking would show a rapid roll-off at 90% relative dose, comparable to the static dose. In reality, the roll-off will shift, dependent on the accuracy of the compensation. For the two test cases, the DAHs confirm the improved coverage. While the uncompensated (no margin) tracking exerts an early roll-off in the shoulder of the DAH, early dose losses can be avoided using the margin expansion. However an early roll-off at about 85% of the relative dose can be observed for both cases. The increased coverage is confirmed in the DAH of figure 10(b). However, the larger evaluation area which includes the dose plateau, reduces the coverage gain.

In table 1, quantitative DAH-parameters extracted from figure 10 are shown. In the shoulder of the reference distribution ($\hat{D}&lt;D&lt;0.97$), the ${{A}_{90 \%}}$ point shows an increase of 47% and 41% for the two on-line experiments when comparing tracking with and without margins. The evaluation over the entire dose plateau ($\hat{D}&lt;D$) shows an improvement in coverage of 14.5% and 11.8%, respectively.

**Table 1.** Quantitative coverage assessment: ${{A}_{90 \%}}$ DAH-parameters of the dose coverage in conventional (index *c*) and margin-compensated (index *m*) tracking.

Volunteer	$\hat{D}&lt;D&lt;0.97$			$\hat{D}&lt;D$		
	${{A}_{90 \%,c}}$	${{A}_{90 \%,m}}$	difference/$ \% $	${{A}_{90 \%,c}}$	${{A}_{90 \%,m}}$	difference/%
1	0.36	0.84	47.36	0.82	0.96	14.47
2	0.51	0.92	41.15	0.86	0.98	11.75

## 4. Discussion

The concept of on-line margin determination could be successfully implemented and tested on real-time hardware, with a mean running time of $22.89\pm 3.46~\text{ms}$. This time included the estimation of the error distribution, margin extraction, segment dilation and segment prescription to the MLC. The experiments show that margin generation adapted to the requirements of a particular motion scenario is feasible.

The on-line motion tracking experiment showed good agreement to the expected coverage improvement with the used reference model. Due to the generally faster inhale velocities, larger errors occur in that direction as compared to the opposing (exhale) direction. The estimator is able to successfully account for these error anisotropies with an accordingly anisotropic margin.

In addition to the dose distribution data, DAH-evaluation showed a significant recovery of the dose within the area over $\hat{D}$ with an increase of ${{A}_{90 \%}}$ of 47.4% and 41.1% in the shoulder and 14.5% and 11.8% in the dose plateau of the reference dose.

Despite the adaptivity and the compliance of the measurements with the theoretical predictions, the results were obviously degraded by an imperfect dose model. This is observed best in figure 9(b), where the shift of aperture causes two effects: firstly, a changed dose plateau, which can be caused by unmodelled changes in scatter behavior of the Linac head. Secondly, the otherwise overlapping 90%-contours in figure 9(a) show imperfect matching at the vertices of the circular aperture. At these points the leaf discretization becomes relevant during tracking, thus producing significantly different dose contours when comparing static and tracked doses.

The depth-dependency of the PGK showed by Low *et al* (1995) is not addressed in this work. Due to the planar measurement, the measurement depth was kept constant for the experiments. However, when tracking targets immersed into a bulk (such as the abdomen), characterization of the depth-dependent penumbra might be prerequisite and its impact should be assessed. It is expected, that the PGK increases slowly with increasing depth (Metcalfe *et al* 1993).

As shown by (Falk *et al* 2010), MLC-tracking enables a significant reduction of safety margins at the planning stage. For residual tracking errors, the auto-adaptive character of the adaptive margin technique enables a generic compensation of induced underdosages, independent of the specific MLC tracking system or patient characteristics. An important constraint, however, is the validity of the training data within a unique set of machine and target geometry. If this correlation changes (e.g. by rotating the gantry, MLC angle), the training data is invalidated and the margin generator has to be retrained for the particular BEV. Another way to approximate stable training data is to change the machine-target geometry slowly enough to approximate a quasi-static transition. This could be performed e.g. by setting a fixed MLC angle along the main direction of displacement due to breathing, i.e. in caudo-cranial (CC) direction. This implementation can be found in the Elekta MR-linac (Lagendijk *et al* 2014) and keeps the main motion axis parallel to the MLC leaf-travel direction. Accordingly, error statistics of the axis where the largest margin is applied can be considered to change very slowly (quasi-statically) with moving BEV. Although, the compensation of geometric error is only restricted by the field size of the MLC, additional safety interlocks should be triggered, once the geometric error exceeds a well defined level.

An important feature of the tracking margin generator concept is its design to retain target coverage based on machine error parameters, not on patient characteristics. The increased target coverage, obtained by the expanded segments, is accompanied by an overdosage in the area outside the original segment. This in turn implies that surrounding organs at risk (OAR), which are potentially spared with high conformality in the planning phase, may receive higher doses than intended due to the segment expansion. Equally, doses higher than 100% can occur in target regions, when multiple segments of e.g. intensity modulated radiotherapy (IMRT) constructively interfere. In order to regularize these effects, leaf shaping algorithms which penalize OAR overdosages (Moore *et al* 2016, Wisotzky *et al* 2016) can be employed.

Intrinsically, despite the interference effects, the overall dose burden to OAR is expected to be significantly lower comparing to non-compensated methods such as the internal target volume (ITV) concept (ICRU 2010). These integral dosimetry measurements should be addressed in future studies evaluating 3D dose distributions for tracked deliveries with and without automatic margin expansion on clinical IMRT-plans, e.g. using a dosimetry phantom such as Bedford *et al* (2015). For practical purposes and because of the superior spatial resolution, film was used in this proof of concept.

The herein described on-line margin generator can be considered as an independent block between aperture prescription and MLC-hardware. If the target motion is predictable, a predictor module will be used to gap the deterministic latencies. Such predictability is exposed by structured motion (Ruan *et al* 2011) caused by regular breathing in the abdomen and thorax. A prediction module potentially increases the gradient in the slopes of the profiles figure 9. The margin generator can then be attached to such a prediction module to correct for the non-deterministic (but stochastic) residual errors.

## 5. Conclusion

We developed and tested a margin generator for tracking error compensation in MLC tracking. The margin generator auto-adaptively imposes a tracking margin in order to retain a desired coverage level. The margin calculation uses statistics based on the patient motion and the ability of the machine to follow these excursions. This enables automatic adaptation to per-patient settings, disregarding tracking margins in the treatment planning stage. The proof-of-concept could show the feasibility of such a strategy. Future work will investigate the impact of this per-segment expansion on a delivered plan. Equally, the margin generator could be coupled with predictor algorithms. This would enable complementary compensation of systematic (predictable) and stochastic (unpredictable) errors.
